# Carrier fluid temperature data in vertical ground heat exchangers with a varying pipe separation

**DOI:** 10.1016/j.dib.2018.04.005

**Published:** 2018-04-06

**Authors:** Nikolas Makasis, Guillermo A. Narsilio, Asal Bidarmaghz, Ian W. Johnston

**Affiliations:** a Department of Infrastructure Engineering, The University of Melbourne, Parkville, Australia; b Department of Engineering, University of Cambridge, Cambridge CB2 1PZ, United Kingdom

## Abstract

The dataset in this article is related to shallow geothermal energy systems, which efficiently provide renewable heating and cooling to buildings, and specifically to the performance of the vertical ground heat exchangers (GHE) embedded in the ground. GHEs incorporate pipes with a circulating (carrier) fluid, exchanging heat between the ground and the building. The data show the average and inlet temperatures of the carrier fluid circulating in the pipes embedded in the GHEs (which directly relate to the performance of these systems). These temperatures were generated using detailed finite element modelling and comprise part of the daily output of various one-year simulations, accounting for numerous design parameters (including different pipe geometries) and ground conditions. An expanded explanation of the data as well as comprehensive analyses on how they were used can be found in the article titled *“Ground-source heat pump systems: the effect of variable pipe separation in ground heat exchangers” (Makasis N, Narsilio GA, Bidarmaghz A, Johnston IW, 2018)* [1].

**Specifications Table**

**Table:** 

Subject area	*Geothermal Energy, Geotechnics, Engineering*
More specific subject area	*Effect of variable (non-fixed with depth) pipe separation in borehole ground heat exchangers*
Type of data	*Figures, MS Excel file*
How data was acquired	*Generated using experimentally validated finite element modelling*
Data format	*Raw, Analysed*
Experimental factors	*Data processed using statistical methods*
Experimental features	*The effect of a variable pipe separation is determined, as well as its sensitivity to important design parameters*
Data source location	*Data maintained in University of Melbourne servers/databases*
Data accessibility	*Data supplied in article as excel file – supplementary data*
Related research article	*N. Makasis, G.A. Narsilio, A. Bidarmaghz, I.W. Johnston, Ground-source heat pump systems: the effect of variable pipe separation in ground heat exchangers, Comput. Geotech. **100** (2018), 97–109.*

**Value of the data**

• The data were generated using complex and computationally expensive numerical methods (requiring several months to be completed) and can be of use to researchers that are interested in modelling the heat transfer process in shallow geothermal systems.
• The data allow researchers to further expand on the analyses presented in [1] and/or use other statistical tools in investigating this effect.
• The range of input parameters used to generate the data allow researchers to investigate different aspects of this technology and the importance of these parameters.
• The dataset that incorporates randomness can lead to a more extensive statistical analysis of the impact of randomness on these systems.

## 1 Data

The data describe the operation of vertical ground heat exchangers (GHE), used in shallow geothermal technologies to provide efficient and renewable heating and cooling to buildings. A modelled GHE consists of a borehole that has pipe loops embedded within it and transfers heat to and from the ground. The data of this article describe the daily average temperature of the circulating (carrier) fluid in the pipes (average of inlet and outlet) as well as the inlet temperature from which the outlet and the thermal load over time can be derived. These temperatures result from the simulated annual operation of various geothermal systems, each having different design parameters and/or ground conditions, ensuring a large number of annual datasets that satisfy a wide range of possible designs and/or locations. The data provide insights regarding the performance and efficiency of shallow geothermal systems.

## 2 Experimental design, materials, and methods

The data were generated using a validated finite element numerical methodology, and applied through the software COMSOL Multiphysics, which was developed at the University of Melbourne [2], [3], [4]. This methodology models the conduction and convection heat transfer processes taking place during the operation of a shallow geothermal system (see for example [5], [6] for a detailed description of these systems). The simulation process is very computationally expensive and to generate this rich dataset of over 300 simulations, high performance computing was utilised over the course of several months.

**Fig. 1:**
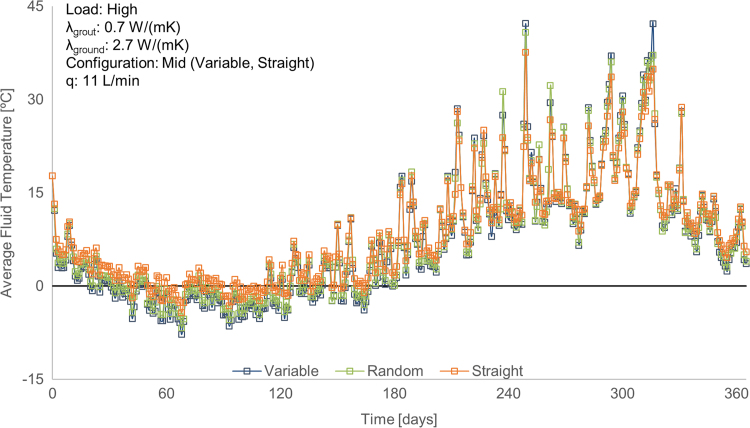
Average fluid temperature for each day of one year simulation, for the three different pipe separation geometries. (Note: Random id = 102).

**Fig. 2:**
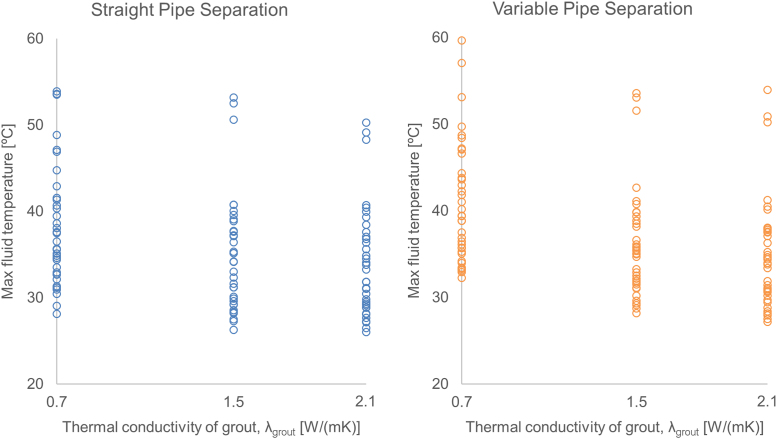
Annual maximum fluid temperature over the thermal conductivity of the grout, for the straight (fixed) and variable pipe separation geometries.

**Fig. 3:**
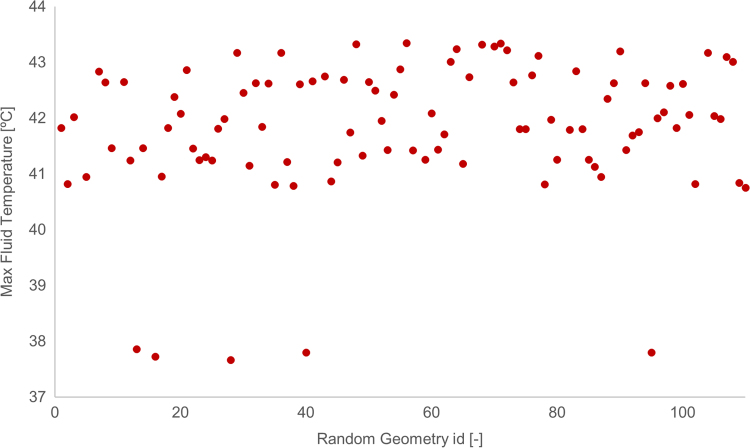
Annual maximum fluid temperature for 100 different randomised pipe geometries.

For each simulation, different design parameters and conditions were used. Overall, the data are separated into three main categories, based on the geometry of the pipes within the GHE, describing the potential effect on the system performance when the placement of the pipes along the depth of the GHE is not as expected or varies. The first category describes the pipes being fixed in place along the depth of the GHE (labelled “Straight”), the second category describes the pipes moving in a sinusoidal pattern along the depth of the GHE (labelled “Variable”) and the third category describes the pipes moving randomly along the depth of the GHE (labelled “Random”). Fig. 1 shows a comparison between these three geometries for one sample set of input parameters, while Fig. 2, Fig. 3 show sample plots that can be created from the supplied data to extract insights. Fig. 2 compares the straight and sinusoidal varying geometries (category 1 and 2) in comparison to one of the design parameters (thermal conductivity of the grout in the GHE, *λ*_grout_) and Fig. 3 demonstrates the design inaccuracy due to randomness in the pipe geometry (category 3). It should be noted that for the case where the pipes move randomly, around 100 different variations of this random geometry were used (seen in x axis of Fig. 3), all using the same set of input parameters. The exact geometries and parameters used to generate the dataset as well as expanded analyses on this topic can be found in [1].
